# Inflammatory and Fibrotic Mediators in Renal Diseases

**DOI:** 10.1155/2019/7025251

**Published:** 2019-02-10

**Authors:** Sandra Rayego-Mateos, Roel Goldschmeding, Marta Ruiz-Ortega

**Affiliations:** ^1^Vascular and Renal Translational Research Group, Institut de Recerca Biomèdica de Lleida (IRBLleida), Lleida 25198, Spain; ^2^University Medical Center Utrecht, Utrecht, Netherlands; ^3^Cellular Biology in Renal Diseases Laboratory, Universidad Autónoma Madrid, IIS-Fundación Jiménez Díaz, Madrid, Spain

Chronic kidney disease (CKD) is a progressive disease that represents a public health problem in our modern society affecting 5-7% of the world's population. Recent clinical studies have described that CKD is an independent risk factor for cardiovascular disease. CKD patients have a 10 to 100 times higher risk than healthy people of developing cardiovascular diseases, including hypertension, diabetes, and hyperlipidemias, all of them associated with high morbidity and mortality. CKD is characterized by a progressive destruction of the renal parenchyma, sustained inflammation, and a functionality loss of the nephron that leads to end-stage renal disease (ESRD). The dysfunction of the nephron triggers cellular and molecular processes that seek to promote the compensatory growth of the nephron. In certain cases, this compensatory mechanism becomes pathological, contributing to renal damage progression leading to ESRD. Nowadays, one of the biggest problems in nephrology is the increased number of patients who progress up to ESRD. These patients require, in 100% of cases, renal replacement therapy such as dialysis (hemodialysis or peritoneal dialysis) or kidney transplantation. Importantly, 50% of patients who undergo dialysis, cannot stand more than three years with these replacement therapies, hence the importance of developing new strategies that could restore kidney function or, at least, delay disease progression.

This special issue contains 6 papers, including original research articles, clinical research articles, and a review, ranging from detailed studies of the key molecular mechanism associated to inflammation and fibrosis in renal diseases.

In the clinical field, there is a need for more studies that can deepen in the identification of new biomarkers for early detection and prevention of kidney disease progression. The manuscript entitled “Circulating CD14^+^CD163^+^CD206^+^ M2 Monocytes Are Increased in Patients with Early Stage of Idiopathic Membranous Nephropathy” by J. Hou et al. studied the changes in peripheral blood monocytes and their clinical significance in patients with an early stage of idiopathic membranous nephropathy (IMN). Approximately 30–40% of patients with this common type of nephrotic syndrome progress to CKD. Recent studies have shown the importance of the differentiation of macrophages in the pathogenesis of chronic inflammatory process, including CKD. Monocytes are the progenitors of dendritic cells or macrophages in the tissues. The ratio of M1/M2 monocytes/macrophages is known to be modified in several kidney diseases but this has not been studied in detail for IMN. M1 monocytes release proinflammatory cytokines such as IL-12 (Th1 immune response), while M2 monocytes secrete anti-inflammatory mediators such as IL-10 (Th2 immune response) and have been suggested to exert profibrotic actions. In this manuscript, the authors show that CD14+CD163+CD206+ M2 monocytes are relevant for the pathological process in incipient disease and are correlated with the severity of the disease. The authors also propose that IL-10^+^ M2 cells should be a useful marker for evaluating the severity of incipient IMN. Cell-based therapy related to M2 macrophages is an active field of research. The phenotype and function of macrophages at the different CKD stages are not well defined yet, since injection of different types of macrophages (bone marrow-derived, splenocyte-derived, or cell line-derived macrophages) in different models of experimental renal damage has revealed opposite results. Therefore, future studies are needed to define possible macrophage-based therapies.

The study by A. Żyłka et al. entitled “Markers of Glomerular and Tubular Damage in the Early Stage of Kidney Disease in Type 2 Diabetic Patients” analyzed several markers of glomerular and tubular damage in patients with type 2 diabetes with these inclusion parameters: eGFR > 60 ml/min/1.73 m2 and uACR < 300 mg/g, and at different stages of CKD (G1/G2, A1/A2). In this study, the authors identified serum cystatin C and urine IgG, transferrin, and NGAL as the best indicators of glomerular damage, whereas urine NGAL, KIM-1, or uromodulin could be indicators of tubular damage. The urinary markers are associated with increase of albuminuria, while both serum and urine NGAL were significantly associated with eGFR decline. Although these findings still need to be confirmed in a large number of patients, these data indicates that combination of biomarkers in serum and urine could be useful for the clinical management of diabetic patients.

Cardiovascular and heart damage is the most frequent consequence of CKD. Serum levels of sST2 (soluble suppression of tumorigenicity 2), a novel biomarker of fibrosis and cardiac remodeling in heart failure patients, are independent of eGFR and age. Previous studies described that the BCN Bio-HF score, an algorithm derived from a real-life cohort, based on soluble ST2 in addition to other predictive serum biomarkers (NT-proBNP and hs-cTnT) and clinical variables/treatments (beta-blockers ARBs/ACEI, statins, and furosemide), could be a useful tool in CV risk stratification of nondialyzed CKD patients. M. Plawecki et al. in their manuscript entitled “sST2 as a New Biomarker of Chronic Kidney Disease-Induced Cardiac Remodeling: Impact on Risk Prediction” evaluate the predictive potential of serum sST2 in CKD patients and show that serum sST2 alone is not a good predictive biomarker of major adverse coronary events or death in CKD patients.

Y. Xu et al. in their manuscript entitled “Apoptosis-Associated Speck-Like Protein Containing a CARD Deletion Ameliorates Unilateral Ureteral Obstruction Induced Renal Fibrosis and Endoplasmic Reticulum Stress in Mice” showed the key role of the apoptosis-associated speck-like protein containing a CARD (ASC), a component of the inflammasome, in the progression of interstitial fibrosis in a model of experimental renal damage. The genetic deletion of ASC in mice submitted to unilateral ureter obstruction significantly reduced the inflammatory cell infiltration in the kidney. The ASC knockout mice showed a diminution in apoptosis (lower number of TUNEL-positive cells, decreased levels of Bax and caspase 3, and increased levels of Bcl-2) and in fibrosis assessed by the expression of ECM proteins, such as fibronectin and collagen I. Additionally, they demonstrated improvement of endoplasmic reticulum stress as evidenced by the reduced BIP, p-eIF2*α*/eIF2*α*, ATF4, and CHOP protein levels in the obstructed ASC-deleted mice compared to obstructed WT mice. These results demonstrated the key role of the ASC inflammasome component in the regulation of the inflammatory and fibrotic processes in experimental renal damage.

Another study submitted to this special issue by K. Ramani et al. is entitled “IL-17 Receptor Signaling Negatively Regulates the Development of Tubulointerstitial Fibrosis in the Kidney.” In this study, the authors showed the crucial role of the IL-17A signaling pathway in the modulation of renal fibrosis induced by comparing fibrosis upon ureteral obstruction in the Il17ra genetic deleted as compared to wild-type mice. Interestingly, this study demonstrated the novel relationship between the antifibrotic kallikrein-kinin system (KKS) and the IL-17A signaling pathway in the kidney. Interestingly, they found that treatment of the more fibrosis-prone Il17ra-/- mice with bradykinin, the major end-product of KKS activation, confers protection against fibrosis by upregulating the expression of matrix degrading enzymes such as the metalloproteinase MMP2. Moreover, the group identified the bradykinin receptors Bdkrb2 and Bdkrb1 as downstream mediators of IL-17-KKS-axis and administration of a bradykinin receptor 1 or 2 agonist in vivo showed a clear diminution of renal fibrosis.

Finally, the review article from S. *Rayego-Mateos* et al. entitled “Role of Epidermal Growth Factor Receptor (EGFR) and Its Ligands in Kidney Inflammation and Damage” describes the key role of the EGFR signaling pathway in the pathogenesis of inflammatory renal damage, with special attention to the role of some EGFR ligands in this process such as TGF-A and HBEGF that play a crucial role in EGFR transactivation through the modulation of key factors of renal damage and inflammation such as Ang II, aldosterone, and TWEAK. The authors specially analyzed the role of CTGF/CCN2, one recently described EGFR ligand with a strong impact in inflammatory and profibrotic processes in kidney disease.

In summary, this special issue will help to understand the inflammatory and profibrotic mechanisms that regulate key processes in the renal pathology and the new therapeutic strategies that are developed for the treatment and prevention of CKD.

